# Supplementary material for: Soft Mechanical Metamaterials with Transformable Topology Protected by Stress Caching
Source: Adv Sci (Weinh). 2023 May 28;10(22):2302475. doi: 10.1002/advs.202302475 (PMC10401159; doi:10.1002/advs.202302475)
Supplement: Supplementary file 1 — Supporting Information [file ADVS-10-2302475-s005.pdf]

ADVANCED SCIENCE
Open Access

Supporting Information

for *Adv. Sci.*, DOI 10.1002/advs.202302475

Soft Mechanical Metamaterials with Transformable Topology Protected by Stress Caching

Jason Christopher Jolly, Binjie Jin, Lishuai Jin, YoungJoo Lee, Tao Xie, Stefano Gonella, Kai Sun, Xiaoming Mao and Shu Yang**

Supplementary Information

Soft mechanical metamaterials with transformable topology protected by stress caching

Jason Christopher Jolly[1†], Binjie Jin[2†], Lishuai Jin[1], YoungJoo Lee[2], Tao Xie[2], Kai Sun[3], Stefano Gonella[4], Xiaoming Mao[3*] and Shu Yang[1]*

[1]Department of Materials Science and Engineering, University of Pennsylvania, 3231 Walnut Street, Philadelphia, PA 19104, United States.

[2]State Key Laboratory of Chemical Engineering, Department of Chemical and Biological Engineering, Zhejiang University, 38 ZheDa Road, Hangzhou, 310027, Zhejiang, China.

[3]Department of Physics, University of Michigan, 450 Church Street, Ann Arbor, 48109, Michigan, USA.

[4]Department of Civil, Environmental, and Geo-Engineering, University of Minnesota, 500 Pillsbury Drive S.E., Minneapolis, 55455, Minnesota, USA.

*Correspondence to: shuyang@seas.upenn.edu (S. Yang) and maox@umich.edu (X. Mao)

[†]These authors contributed equally to this work.

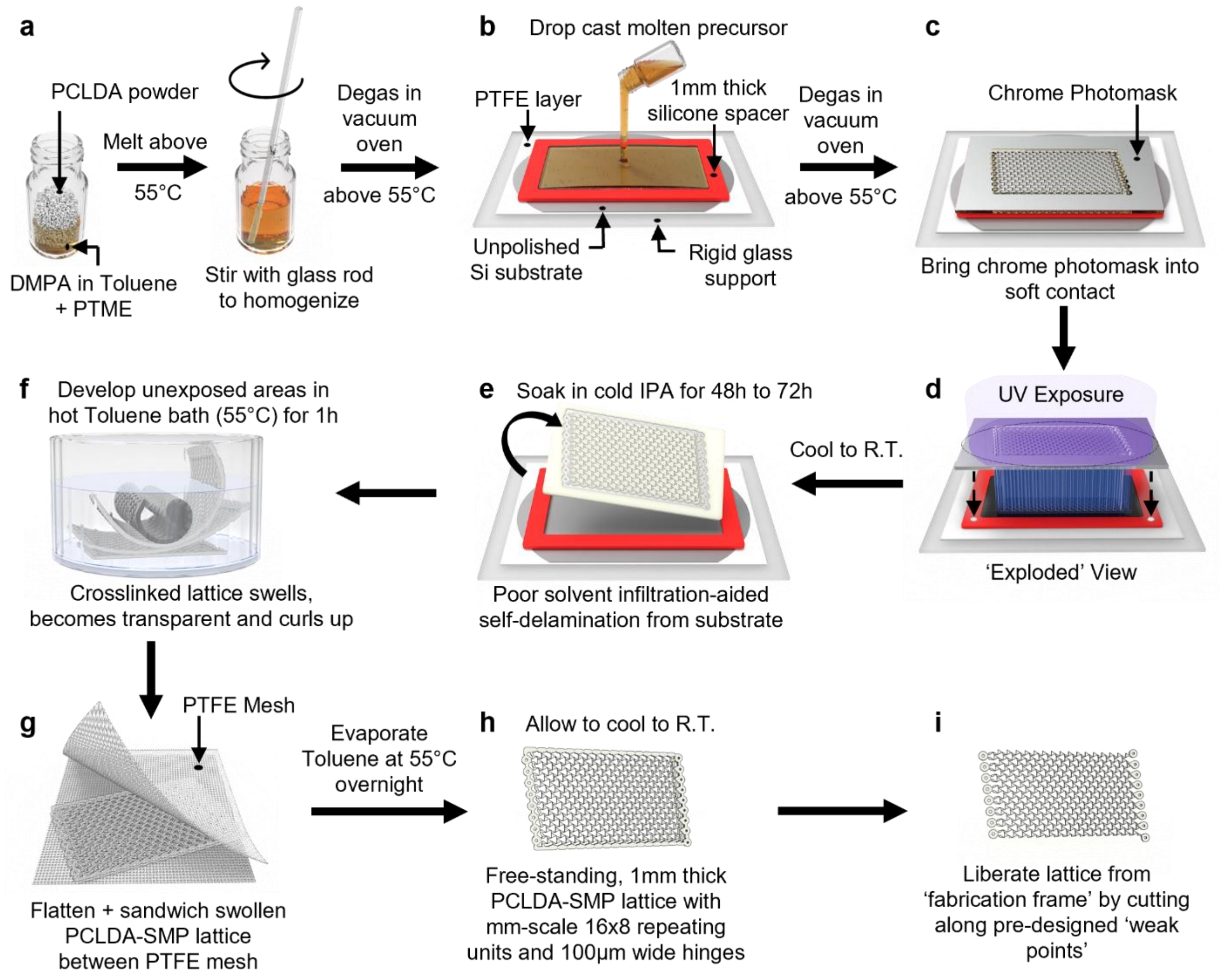

Figure S1 | Fabrication of a free-standing PCLDA-SMP TTMM lattice via a multi-step 'thick' photolithography process.

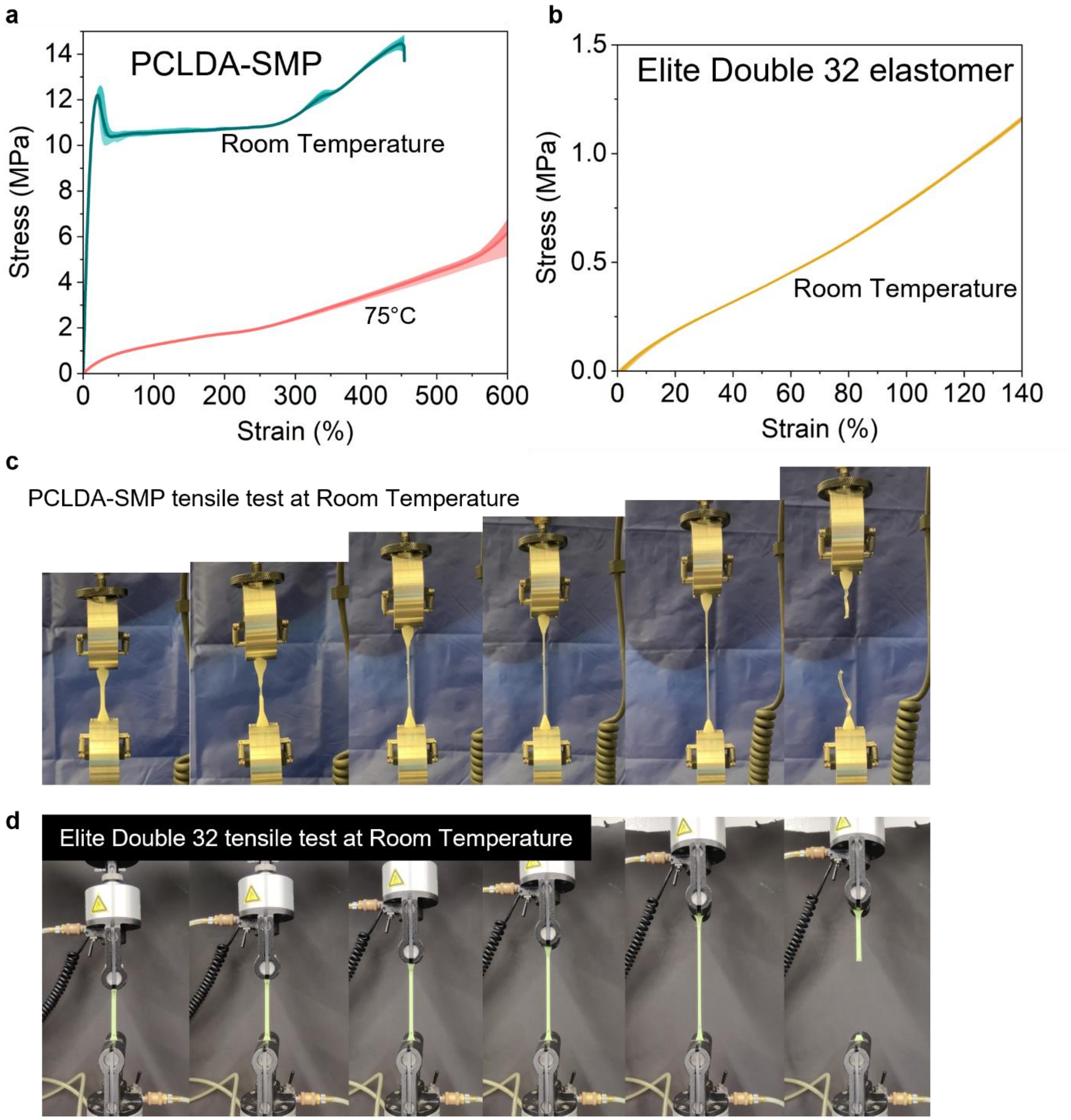

Figure S2 | a,c) Tensile test results of PCLDA-SMP dog bone samples both at room temperature and above its melting temperature and b,d) Tensile test results of Elite Double 32 dog bone samples at room temperature.

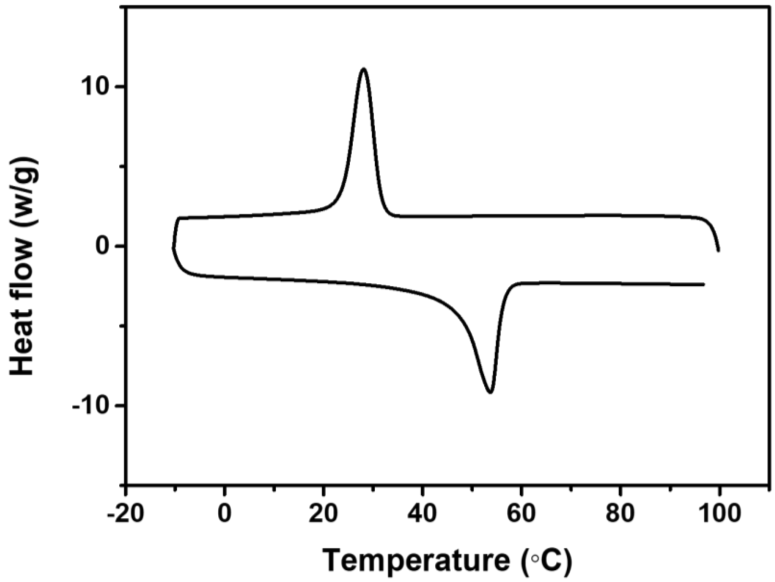

Figure S3 | Characterization of the melting and crystallization transition temperatures of PCLDA-SMP via Differential Scanning Calorimetry (DSC).

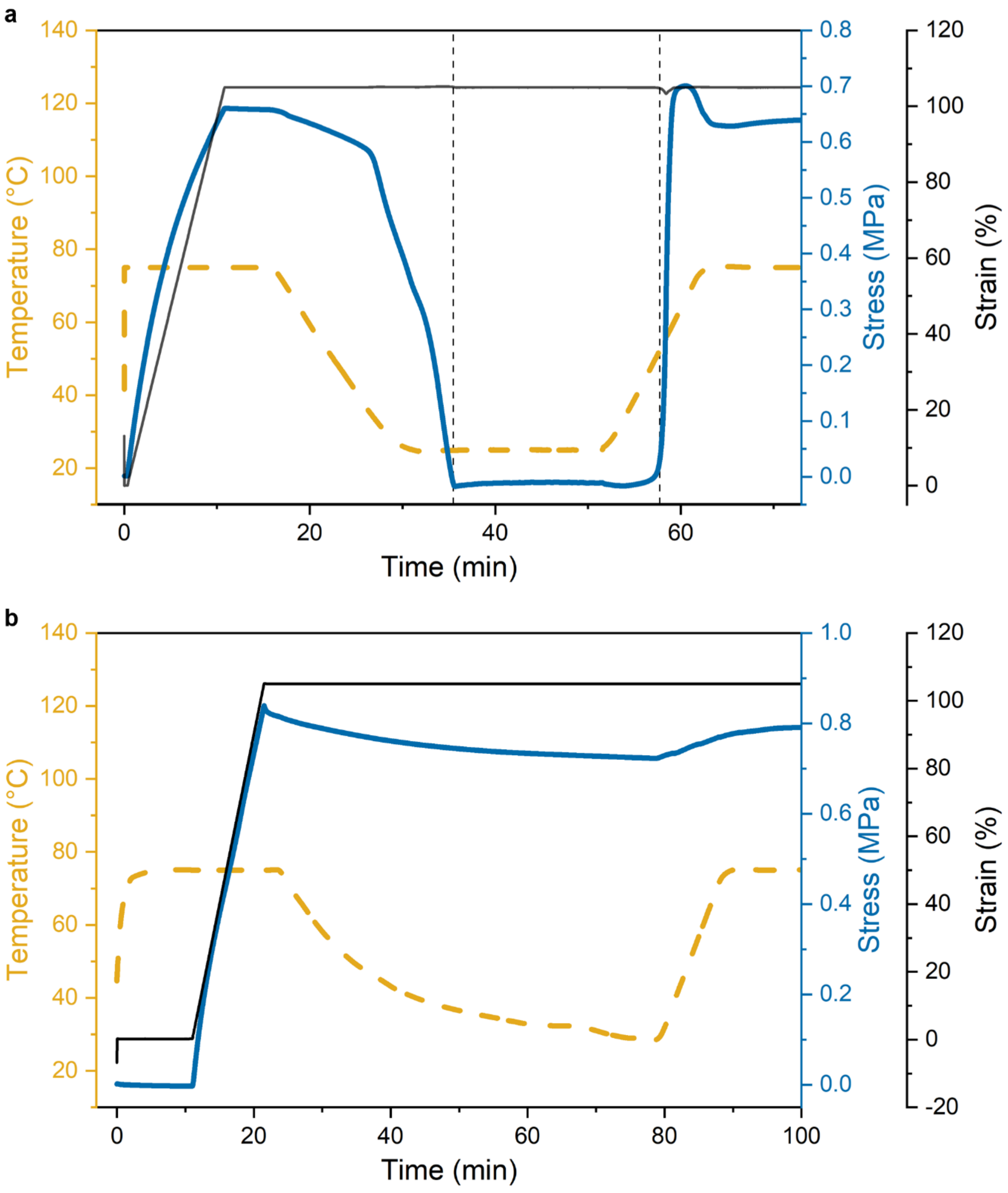

Figure S4 | a) Characterizing the stress caching ability of PCLDA-SMP via Dynamic Mechanical Analysis (DMA) i.e., its ability to 'lock away' any stresses generated upon stretching (to a maximum strain of 105%), upon cooling below its melting temperature and to maintain a 'zero residual stress state' while at room temperature. These restorative elastic stress are unlocked upon reheating above the melting temperature. b) A similar DMA test performed on an Elite Double 32

elastomer sample which serves as a reference case for a polymer system without stress caching, wherein nearly all generated stress remains ‘unlocked’, always.

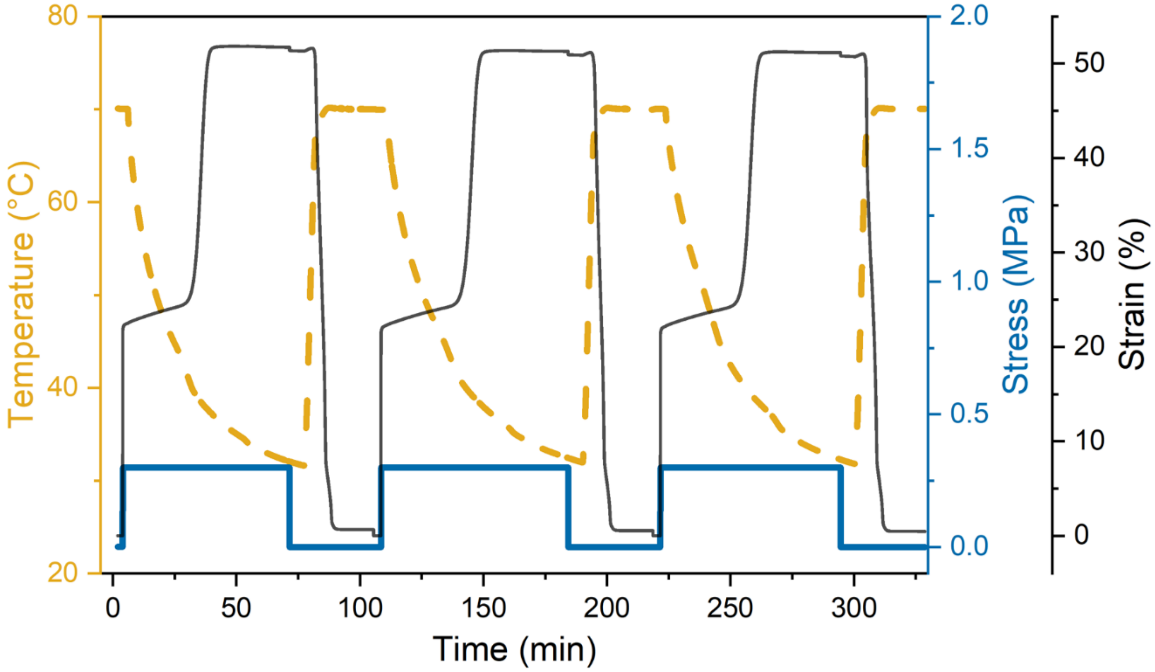

Figure S5 | Characterizing the robustness and repeatability of the intrinsic shape memory mechanism of PCLDA-SMP via Dynamic Mechanical Analysis (DMA) over 3x cycles.

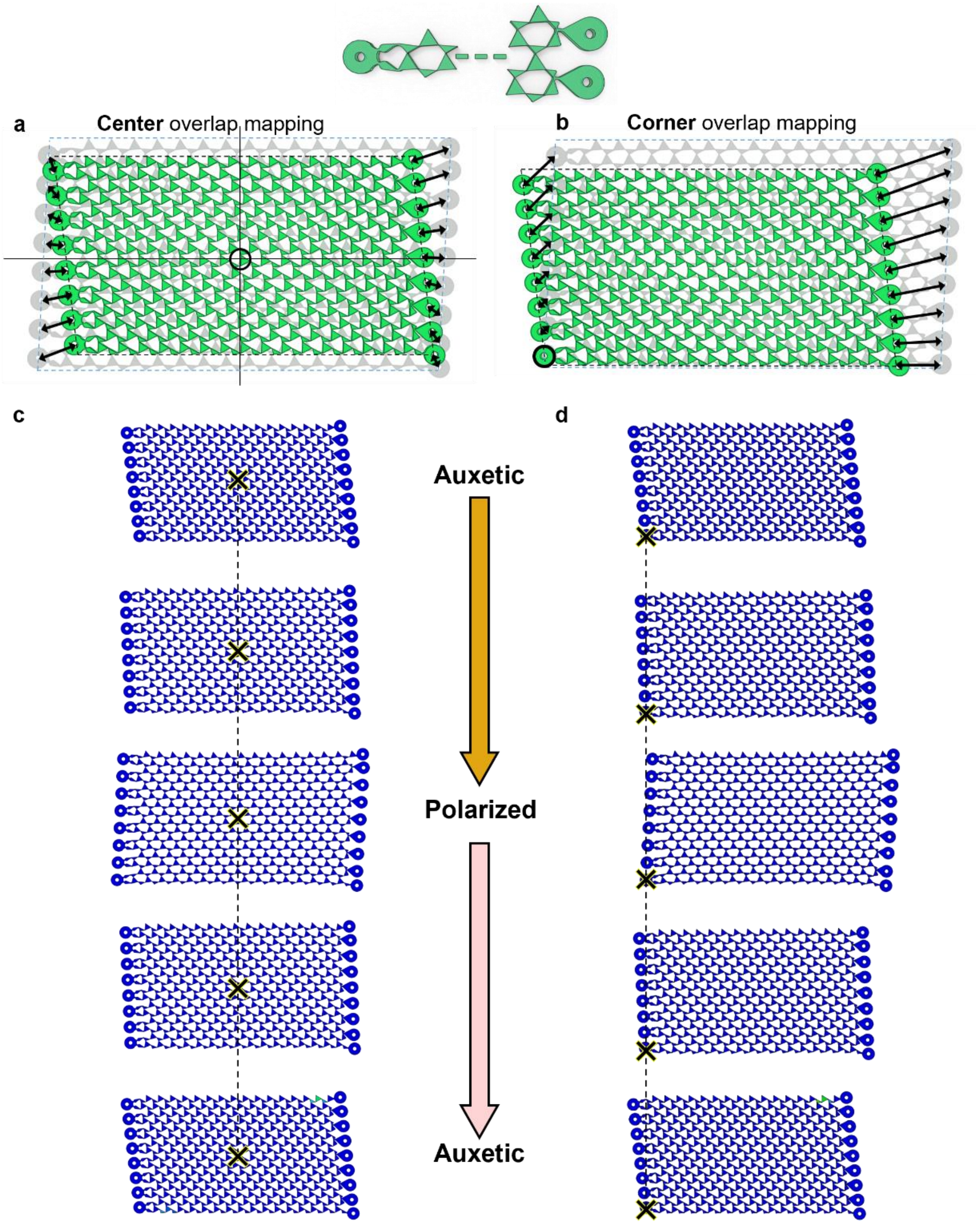

Figure S6 | Mapping the edges of initial and final lattice configurations determines the vector displacements required to prescribe kinematic transformations. a,b) Overlaying auxetic and polarized variants about (a) their centers and (b) one of their corners, permits the definition of vectors that map the corners of triangular units or centers of edge loops along 'input' edges to their equivalent positions in a transformed lattice. c,d) Snapshots of simulation results that confirm the equivalence of kinematic transformations with vectors defined using different common overlay

points, in prescribing a reversible transformation of the same lattice between the same two auxetic and polarized states.

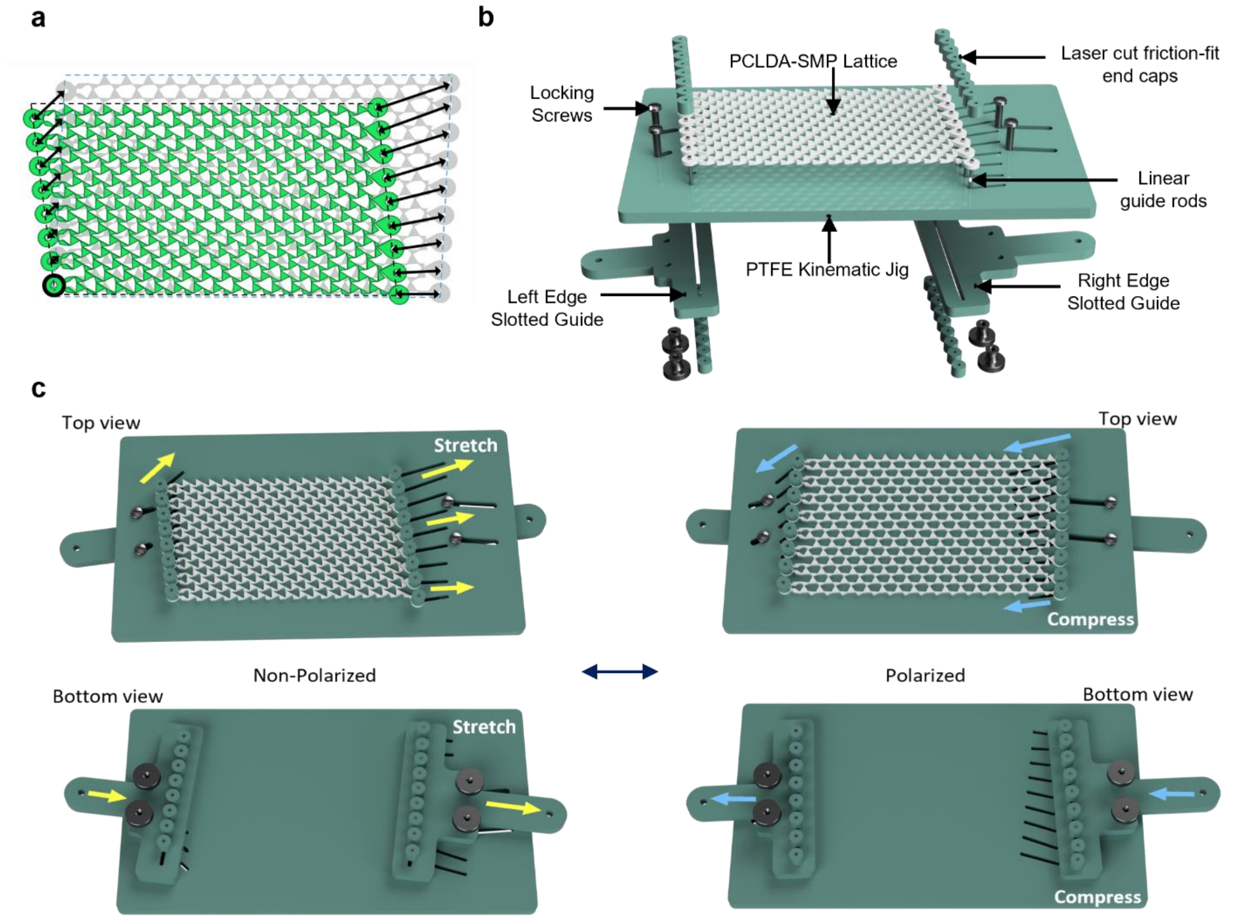

Figure S7 | Design and assembly of a K-2 PCLDA-SMP lattice and its kinematic jig. a) The vector inputs and by extension, the design of the slots to be cut into the kinematic jig, are obtained by overlaying auxetic and polarized lattice conformations about a shared corner. b) Assembling the various components of the kinematic jig and the PCLDA-SMP lattice. c) Schematic of the lattice being transformed between its auxetic and polarized phases upon vectored stretching and compression in the physical jig, respectively.

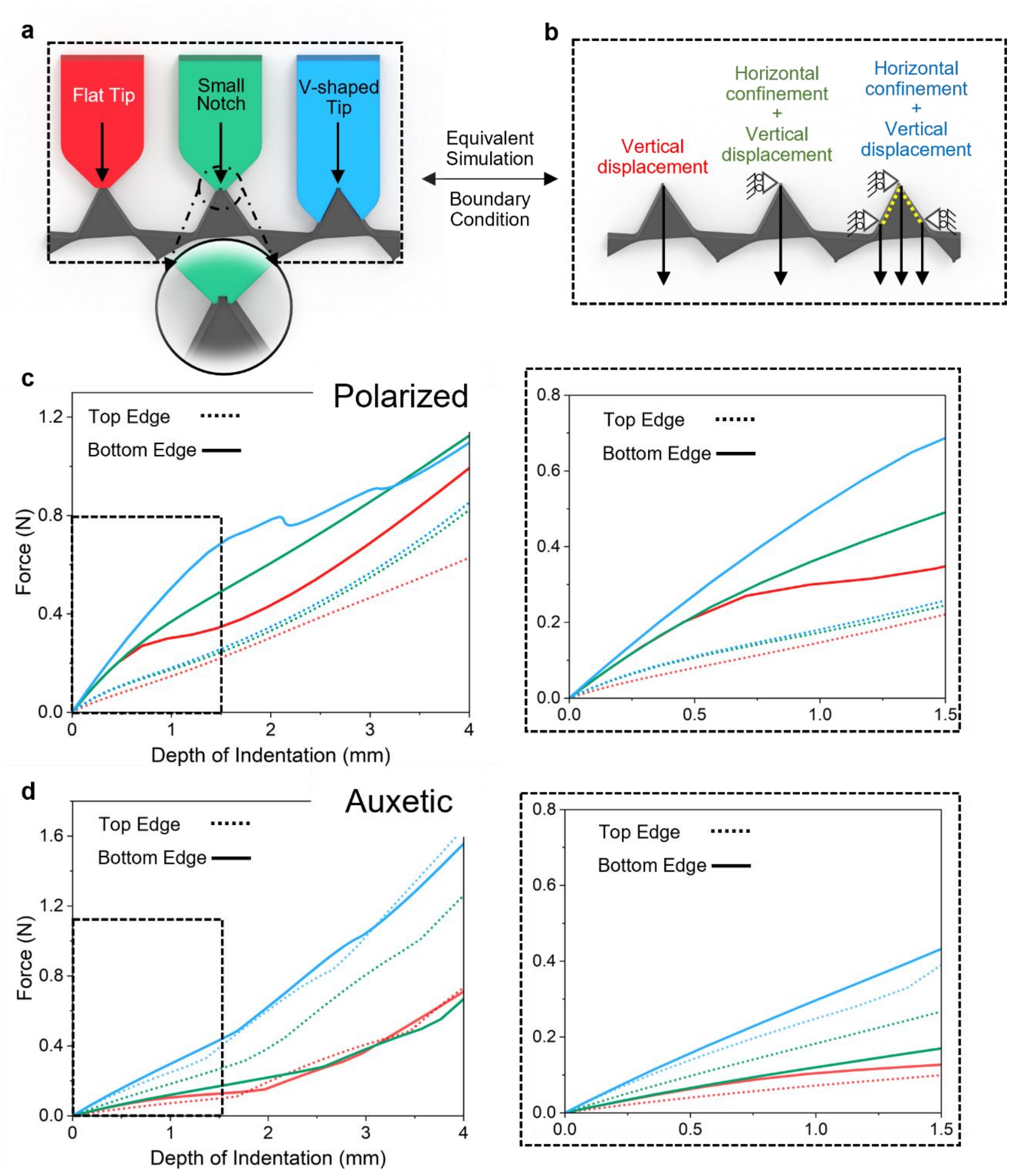

Figure S8 | Effect of indenter tip geometry on topological edge behaviour. a) Three different indenter tip geometries and their influence on mechanical edge behavior were studied via FEM. b) Their respective interactions with the lattice were simulated as different boundary conditions at the tip and edges of the local triangular unit being indented. c,d) Force-displacement curves obtained by simulating the indentation of (c) polarized and (d) auxetic lattices with the aforementioned indenter tip geometries.

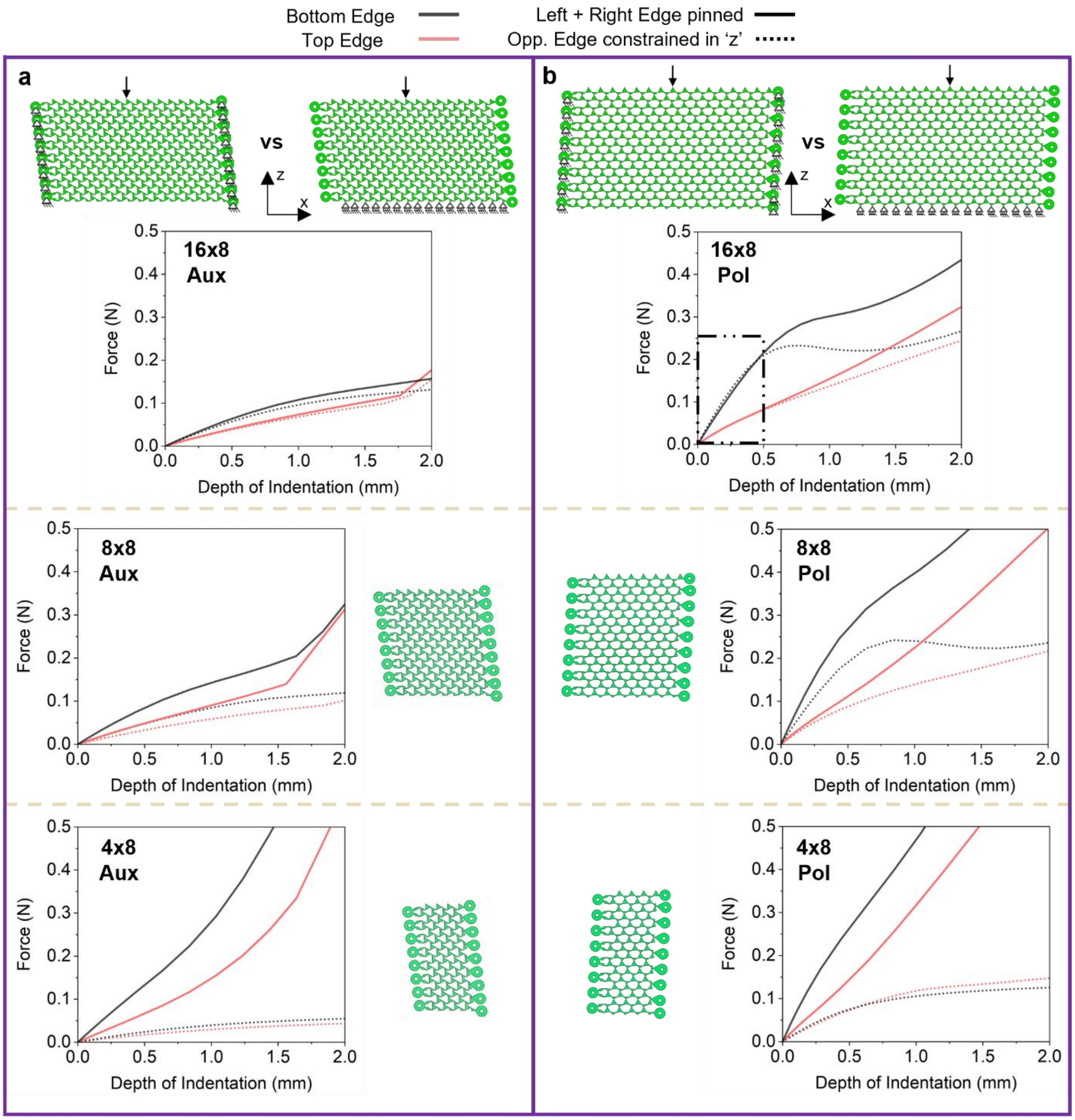

Figure S9 | a,b) Effect of sample boundary conditions during indentation i.e., L-R edge pinning versus bottom edge pinning, on the topological behavior of three different sizes of (a) auxetic lattices and (b) polarized lattices.

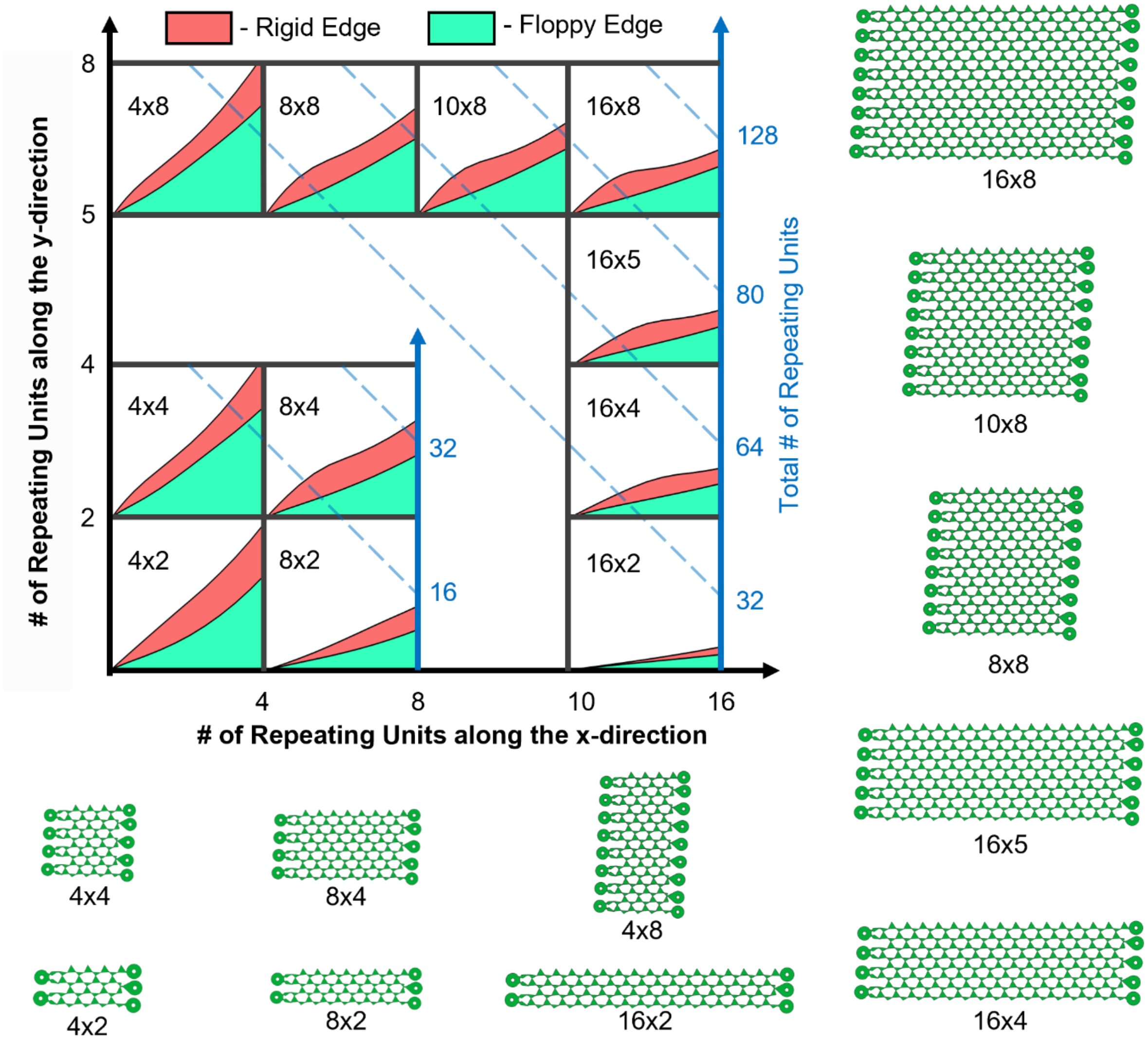

Dimensions of each square sub-plot: 2 mm (Depth of Indentation) x 1 N (Force)

Figure S10 | Effect of lattice size and aspect ratio on topological edge behavior

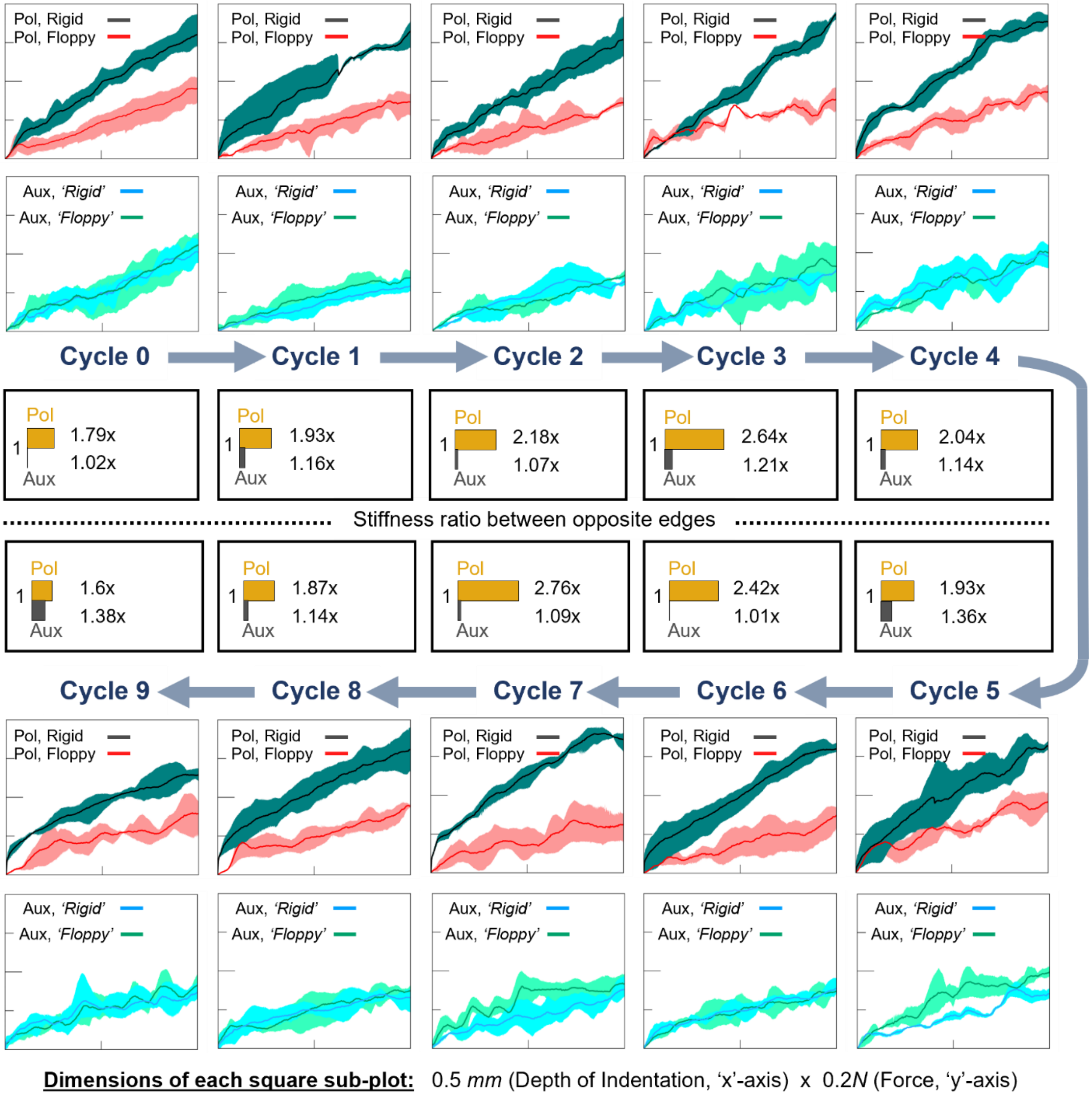

Figure S11 | Edge indentation tests on auxetic and polarized configurations of the same PCLDA-SMP TTMM Lattice subjected to 10x complete kinematic cycles. Stiffness ratios (see histograms) were calculated from the slopes of the linear, small indentation regimes. The results reveal good cyclability between auxetic (S.R.~1.15) and polarized phases (S.R.~2.1)

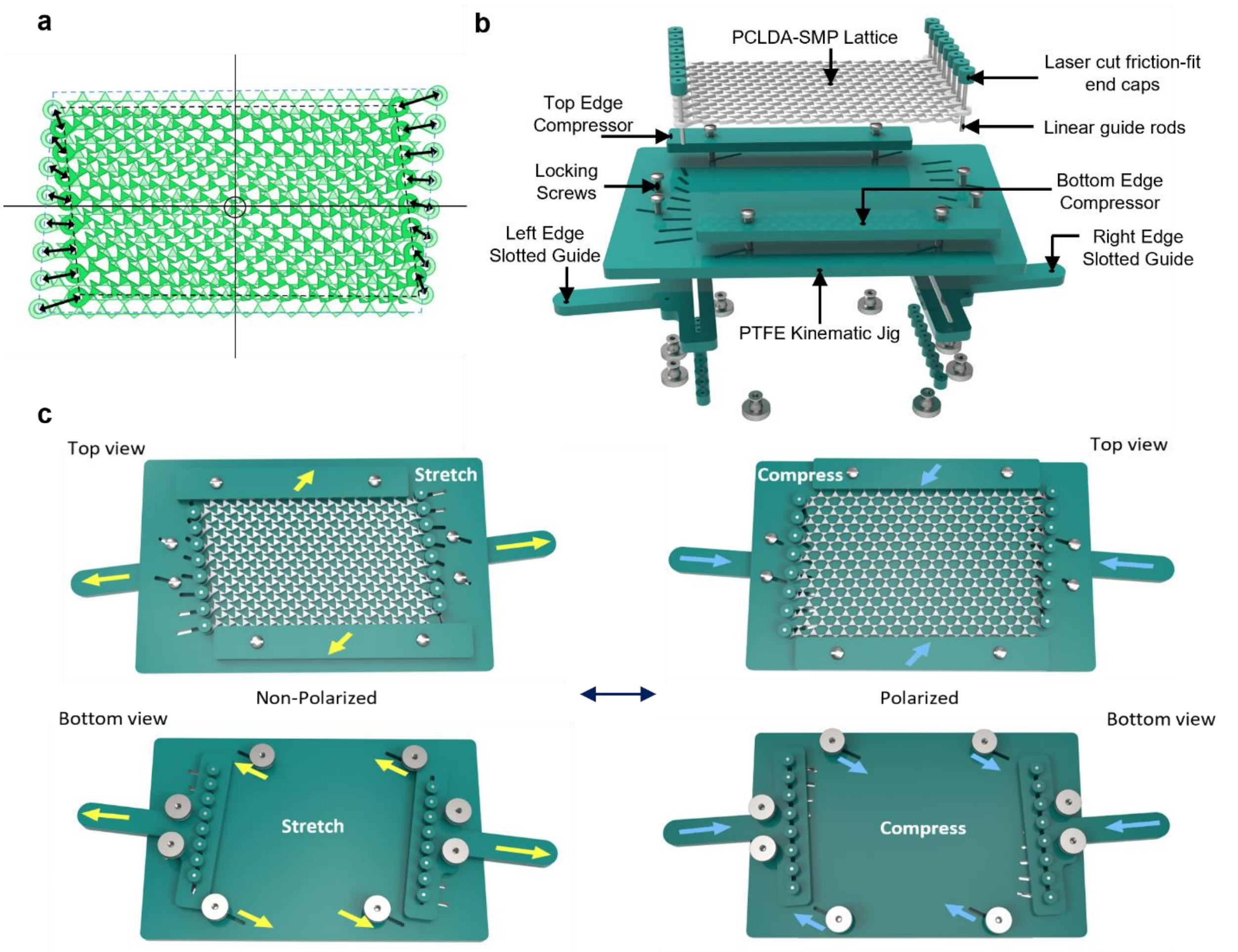

Figure S12 | Design and assembly of a K-1 PCLDA-SMP lattice and its kinematic jig. a) The vector inputs and by extension, the design of the slots to be cut into the kinematic jig, are obtained by overlaying auxetic and polarized lattice conformations about a shared geometric center. b) Assembling the various components of the kinematic jig and the K-1 lattice. c) Schematic of the lattice being transformed between its auxetic and polarized phases upon vectored stretching and compression in the physical jig, respectively.

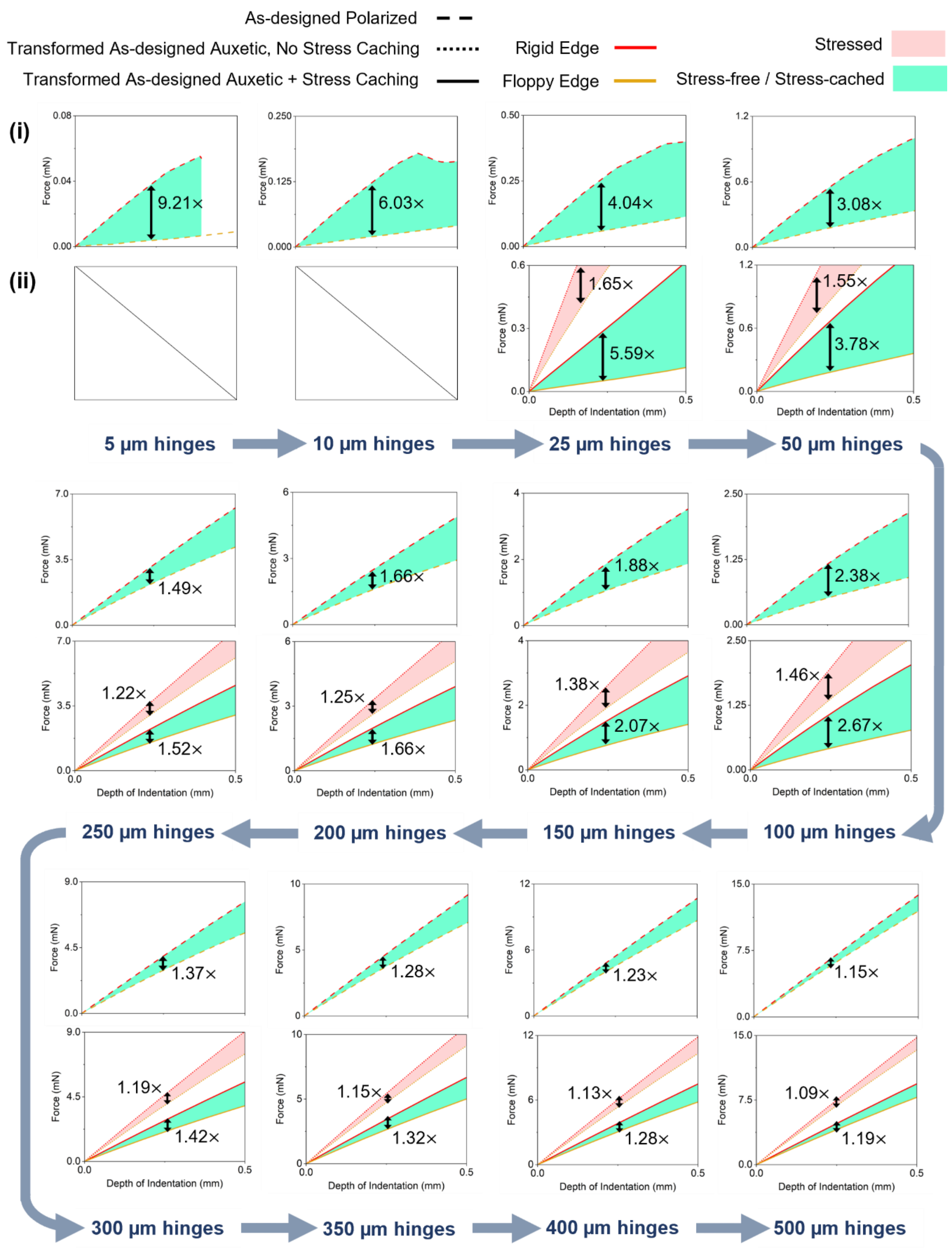

Figure S13 | Simulated indentations on lattices with systematically varying hinge width and with material properties representative of the commercial elastomer (ED-32). i, ii) Force-displacement curves and stiffness ratios of (i) as-designed polarized lattices and (ii) as-designed auxetic

structures that have been stretched into their polarized configurations, before and after stress caching. Simulations utilized the Gent hyperelastic model to capture the behavior of ED-32.

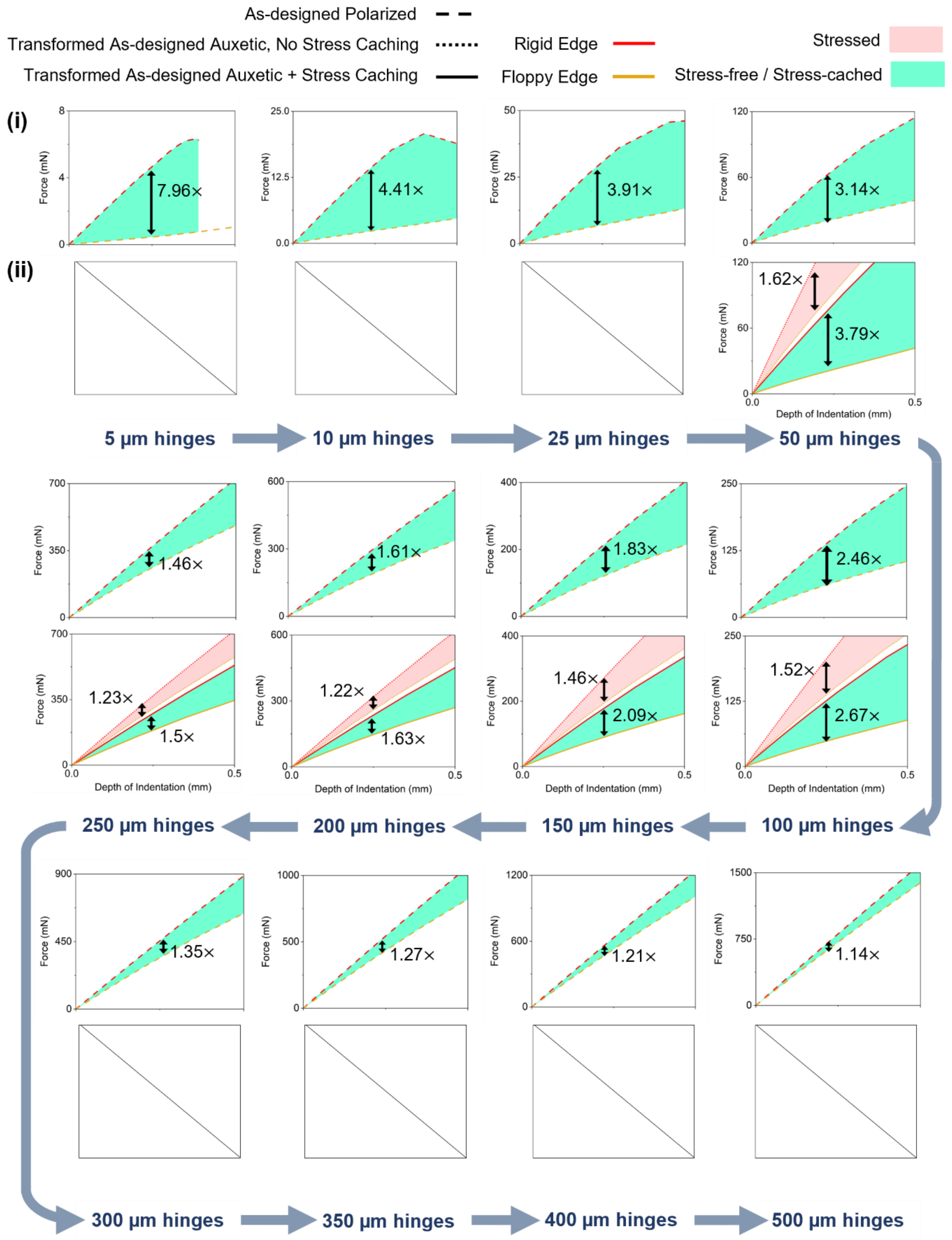

Figure S14 | Simulated indentations on lattices with systematically varying hinge width and with material properties representative of PCLDA-SMP. i, ii) Force-displacement curves and stiffness

ratios of (i) as-designed polarized lattices and (ii) as-designed auxetic structures that have been stretched into their polarized configurations, before and after stress caching. Simulations utilized an elastic-plastic model to capture the behavior of PCLDA-SMP.

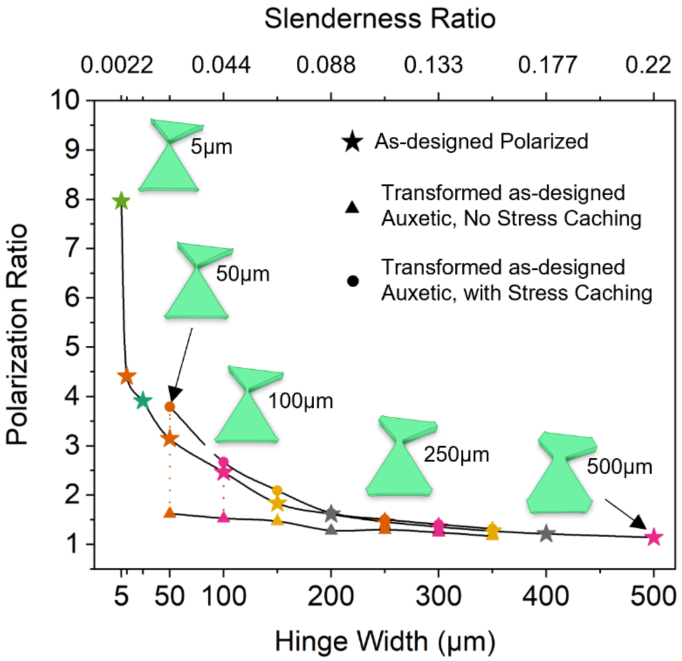

Figure S15 | Simulated effects of hinge slenderness on topological polarization in a stiff, elastic-plastic material i.e., utilizing the room temperature mechanical properties of PCLDA-SMP.

Supplementary Movies

Supplementary Movie 1

Finite element simulation results verifying the effectiveness of the proposed kinematic strategy in prescribing a reversible transformation between topologically distinct phases via a set of synchronously applied vector displacements of lattice edge loops.

Supplementary Movie 2

Finite element simulation results of different-yet-equivalent strategies to determine a vector map that prescribes a reversible kinematic transformation of a TTMM between its auxetic and polarized phases.

Supplementary Movie 3

Experimental demonstration of a complete kinematic cycle of a TTMM lattice via a custom, laser cut Teflon jig that cascades solitary mechanical inputs applied at the L-R sample edges, into a set of synchronously-applied yet individually-prescribed vector displacements of each edge loop. This in turn induces a wholly-determinate biaxial global transformation of the TTMM into a targeted topological phase, courtesy the lattice's Guest Hutchinson mode.

Supplementary Movie 4

Experimental characterization of the topological edge behavior of a TTMM lattice via quasi-static mechanical indentation of opposite 'output' edge pairs i.e., top and bottom edges.

Supplementary Movie 5

Experimental observations of un-cached elastic stresses generated in lattice hinges during a kinematic transformation causing a polarized lattice to ‘snap back’ to its as-fabricated unstressed state. This is only seen in the absence of shape memory and stress caching. Lattices made from a shape memory polymer such as PCLDA-SMP, are stable in any transformed state courtesy their intrinsic ability to cache stresses.
